# Deriving equivalent symbol-based decision models from feedforward neural networks

**DOI:** 10.3389/frai.2025.1618149

**Published:** 2025-07-24

**Authors:** Sebastian Seidel, Uwe M. Borghoff

**Affiliations:** ^1^KNDS Deutschland GmbH & Co. KG, Munich, Germany; ^2^Institute for Software Technology, University of the Bundeswehr Munich, Neubiberg, Germany

**Keywords:** artificial neural networks, connectionism, decision trees, explainable AI, symbolic AI models, symbolism

## Abstract

Artificial intelligence (AI) has emerged as a transformative force across industries, driven by advances in deep learning and natural language processing, and fueled by large-scale data and computing resources. Despite its rapid adoption, the opacity of AI systems poses significant challenges to trust and acceptance. This work explores the intersection of connectionist and symbolic approaches to artificial intelligence, focusing on the derivation of interpretable symbolic models, such as decision trees, from feedforward neural networks (FNNs). Decision trees provide a transparent framework for elucidating the operations of neural networks while preserving their functionality. The derivation is presented in a step-by-step approach and illustrated with several examples. A systematic methodology is proposed to bridge neural and symbolic paradigms by exploiting distributed representations in FNNs to identify symbolic components, including fillers, roles, and their interrelationships. The process traces neuron activation values and input configurations across network layers, mapping activations and their underlying inputs to decision tree edges. The resulting symbolic structures effectively capture FNN decision processes and enable scalability to deeper networks through iterative refinement of subpaths for each hidden layer. To validate the theoretical framework, a prototype was developed using Keras .h5-data and emulating TensorFlow within the Java JDK/JavaFX environment. This prototype demonstrates the feasibility of extracting symbolic representations from neural networks, enhancing trust in AI systems, and promoting accountability.

## 1 Introduction

AI has become a focal point of attention, often described as experiencing a “hype”. This surge of interest is driven by its transformative potential across industries, promising significant efficiency gains and driving innovation, particularly in the areas of image recognition and analysis and natural language processing. The proliferation of large datasets, coupled with increasingly powerful computational resources, has further accelerated the development and deployment of AI applications. As a result, AI has become a cornerstone of modern IT systems, embedded in everything from consumer products to large-scale industrial processes. Despite these advancements, a significant gap remains in terms of trust (Chamola et al., 2023) and acceptance for AI systems, particularly those perceived as “black boxes” (Adadi and Berrada, 2018). As the complexity of human-AI interaction increases, recent research has emphasized the importance of formal architectures that support coordination across heterogeneous agents, including humans, LLMs, and specialized AI components (Borghoff et al., 2025b). This work complements these developments by introducing a symbolic framework that interprets the decision logic of neural networks, enabling their integration into structured, transparent, and more trustworthy multi-agent systems (Borghoff et al., 2025a).

This lack of trust has led to a growing interest in XAI. The core goal of XAI is to make AI systems more transparent and interpretable by providing understandable explanations for their decisions and actions (Gilpin et al., 2018). By addressing the opaque nature of many AI algorithms, XAI aims to bridge the gap between the technical intricacies of machine learning and the human need for understanding. Transparency in AI is not only a technical challenge, but also a social and ethical imperative (Sabbatini and Calegari, 2024). Stakeholders need to trust AI systems to interact effectively with them. This is especially critical in sensitive areas where the stakes of incorrect or misunderstood AI decisions are high, such as military applications (Nitzl et al., 2025a,b). It is also important to prevent AI from being increasingly integrated into criminal and harmful activities and going undetected (Blauth et al., 2022).

The present work contributes to this field by offering a systematic categorization and analysis of symbolic and connectionist approaches within AI. Symbolic approaches are grounded in explicit, rule-based reasoning, while connectionist approaches, exemplified by artificial neural networks, rely on distributed representations and statistical learning. This paper not only delineates the relationships, distinctions, and respective advantages of these approaches but also demonstrates the value of deriving equivalent symbolic decision models from neural models. Such derivations enhance transparency and interpretability without compromising the functionality of the underlying AI systems (Zhang et al., 2021).

A special focus is placed on feedforward neural networks (Bebis and Georgiopoulos, 1994), including similar types of neural networks that can be represented in a feedforward form, such as convolutional neural networks. Recurrent architectures are not explicitly considered in this study. The analysis underscores that symbolic models such as decision trees, finite state machines (for classification or as transducers), and behavior trees are viable candidates for representing the decision logic of neural networks. Among these, decision trees are identified as the most appropriate symbolic counterpart due to their fit with internal processes, interpretability, and alignment with the explainability goals of XAI (Dwivedi et al., 2023; Costa and Pedreira, 2023).

This paper thus situates itself within the broader discourse on enhancing AI explainability by bridging the gap between connectionist models and symbolic reasoning. By doing so, it contributes not only to the technical development of interpretable AI systems but also to their societal acceptance and ethical deployment. In the following sections, we delve deeper into the methodologies and findings that underpin this contribution, offering insights into the practical and theoretical implications of deriving symbolic representations for neural networks. This work serves as a step forward in the journey toward making AI not just powerful but also accountable and understandable.

The paper is organized as follows. Section 2 introduces basic concepts and reviews key related work in the field, providing both a solid theoretical framework and practical context. Section 3 outlines the core contribution of this work, detailing the methodology for deriving decision trees from feedforward neural networks (FNNs). The process is demonstrated with clear examples and supported by suitable data structures, broken down into simple, easy-to-follow steps. Notably, the final derivation procedure is also applicable to decision paths in deep feedforward neural networks, ensuring scalability and versatility. Section 4 presents a proof-of-concept prototype capable of managing larger FFNs and CNNs with their components, including different layers, pooling mechanisms, and the hierarchical structure of the resulting decision tree. Additionally, a brief overview of the graphical user interface is provided. Section 5 summarizes the procedure for deriving a hierarchical decision tree from a feedforward neural network, highlighting the creation of decision paths for input vectors and their combination into a unified tree, showcasing how symbol-based models make the neural network's inner workings transparent.

## 2 Basics and related work

Artificial intelligence as a generic term for automated, self-optimizing and similar systems should serve as a starting point for the necessary classification of our subject of investigation. According to Russel and Norvig (2010), the field of artificial intelligence can be divided into the following four categories based on their respective goals:

- realization of human thought
- generating human actions
- realization of rational thought
- generating rational actions

To achieve the last goals, the field of artificial intelligence offers two basic approaches, which are partly contradictory in the way they work. Their competing basic ideas are described by Minsky (1991). The first is the *symbolic approach*, which relies on logic-based formalisms that process information with complete, defined symbols. The second is the *connectionist approach*. This is based on the distributed representation and computation of information using many simple mathematical operations. The associated models are usually represented as networks or as a cascade of vector and tensor computations, as in Abadi et al. (2015), among others. The symbol-based approach corresponds to a top-down procedure in which the entire problem is first analyzed, then broken down into manageable, defined subproblems, and then solved. The connectionist approach, on the other hand, is a bottom-up procedure in which the existing model is given predefined information about the problem to be solved and independently generates a sufficiently accurate approximation of the solution over several iterations (Minsky, 1991).

The top-down method of the symbolic approach has become established in many technical domains due to the high degree of maturity of the various formal representations of knowledge (An et al., 2024). The underlying advantages of this success are, in particular, efficient, systematic search procedures and the reliable management and control of complex configurations of individual elements and complex interactions of subgoals. The disadvantages come into play when the problems to be solved are too unstructured to be described by universally valid axioms, or when these problems are based on analogies and approximations (Seidel et al., 2019).

The bottom-up method of the connectionist approach was only able to establish itself on a larger scale when symbolic approaches reached their limits in areas such as pattern recognition, automatic optimization, and clustering, and when large data and computing resources became available. The reason for this, besides the hardware requirements, was the lack of internal structure and architecture of these approaches, which made them unsuitable for classical problems of higher reasoning. The problem of lack of structure has been mitigated somewhat in recent decades by the development of new special forms of artificial neural networks, as can be seen in Schmidhuber (2014).

**Figure 1:**
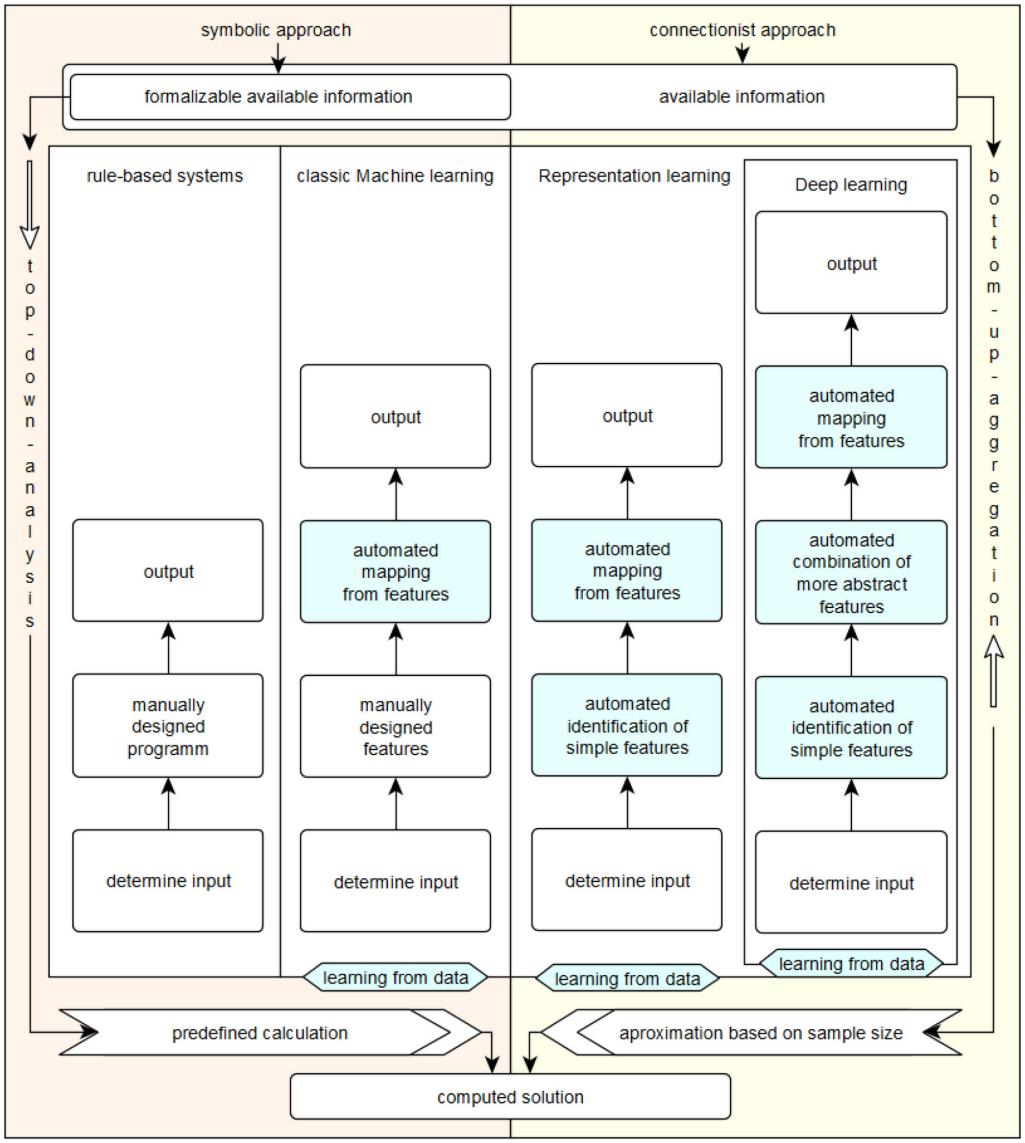
Symbolic vs. connectionist approaches.

Figure 1 summarizes the division into symbol-based and connectionist approaches according to Minsky (1991) and the categorization of the associated methods and systems according to Goodfellow et al. (2016).

*Rule-based systems* include symbol-based approaches, some of which are thousands of years old, such as classical logic. If a problem is simple and can be formalized well, systems in this category can usually solve it reliably and efficiently. This is done by determining the necessary input data, manually constructing an effective procedure for solving the problem, and then determining the desired solution outputs. Examples of methods in this category include inference machines (Browne and Sun, 2001) and reasoning in first-order logic (Bader et al., 2008).

*Classic machine learning* is capable of making assignments on its own based on given, defined characteristics. This category also includes symbol-based approaches. A recent example of this category is decision tree learning, as described in Géron (2022).

It was not until the early 2000s that *learning from representations* could be transferred from theory to an efficiently usable practical application, as described in Schmidhuber (2014). Initially, the input data is determined manually, but then the characteristics of the problem to be solved are determined independently by the system used. Subsequently, the data is independently reorganized and mapped by the respective system on the basis of the determined characteristics in the recognized problem context, and finally the desired solution is displayed. Perhaps the best known example of this category of AI systems are flat artificial neural networks, which are based on the idea of the *perceptron* first described by Rosenblatt (1958). As Goodfellow et al. (2016) explain, connectionist approaches were called cybernetics from 1940 to 1960, and much later deep learning, where the aspect of *depth* of the artificial neural model is not exactly quantified. They refer to multi-layer artificial neural networks as deep neural networks; the term artificial neural networks can be considered as an alternative term for methods and models that learn representations based on the connectionist approach.

*Deep learning* evolved in the late 2000s as the availability of data, storage, and especially computing power continued to increase. This computing power was generated by new hardware, especially graphics cards optimized for vector computations. By providing it in large data centers organized according to the cloud principle, computing power became available more or less independently of location. In addition, motivated by the successes of the early 2000s, a large number of specialized connectionist models were created or old specialized models were made usable in practice. These developments are described in Schmidhuber (2014). The defining feature of deep connectionist models in deep learning is the high number of successive, distributed processing steps. This makes it possible to solve much more complex problems with multiple levels of correlation. However, the aggregation of problem contexts and decision paths is usually beyond human comprehension.

At this point at the latest, questions are being asked about the trustworthiness and explainability of AI (Hagras, 2018; Rawal et al., 2022). Phillips et al. (2021) specify four principles of XAI, see also USgov (2025) and NIST (2023), where the explainability and interpretability of AI models is listed as a relevant property of artificial intelligence. The goal of explainable AI is described among others in Clement et al. (2023) and Turek (2023). Both sources also make clear that the successful use of models from the field of machine learning, and especially those connectionist models from the field of deep learning, makes it necessary to explicitly consider the explainability of AI models.

We will also use the term *artificial neural network* (ANN) for the connectionist approach according to Minsky (1991). Both terms describe the same area of research. ANNs have the ability to learn internal representations for these problems by independently determining and assigning problem characteristics. This usually results in probabilities or probability distributions as a statement about a calculated decision. The internal representations are learned by the corresponding artificial neural networks, which optimize the determination and assignment of features by adjusting their connection weights. To do this, they propagate the deviation of a decision result from the desired result back proportionally through their structure and correct the error portion that each weight had (Rumelhart and McClelland, 1986). The numerical method used for this iterative optimization is usually the gradient descent. For this self-optimization in the event of wrong decisions, there is Dike et al. (2018)

- *supervised learning*, where a suitable output value must be marked or labeled for each individual input when training the network. The provision of data is more complex, but the self-optimization is more reliable and converges faster to a local optimum;
- *reinforcement learning*, where at the end of an individual decision, the only thing that is checked is whether it was useful according to a reward function or not. The weights are then either strengthened or weakened depending on their contribution to the decision. Sufficient data is generated more quickly, but the optimization process is generally less reliable and requires more iterations than supervised learning.
- *unsupervised learning*, where the goal is not to optimize the correctness of a decision, but to optimize the representation for all inputs. A common example is the assignment of inputs with *n* features according to their spatial proximity with respect to these features, e.g. using *k*-means. The data is grouped according to a predefined similarity.

Learned representations of ANNs can be generalized based on similarities (Rumelhart and McClelland, 1986). ANNs are divided into groups of neurons, called layers, which are interconnected. This means that each layer can be viewed as a function of its predecessor layer. Thus, there is a coherent distributed representation of information within each layer. From one layer to its successor layer, the entire distributed representation is modified using a transfer function and, in conjunction with the internal functionalities of the successor layer, forms a new coherent distributed representation. The layered distributed representation of information is reflected in the fact that the locally represented input values in the input neurons of a network in each subsequent layer have a proportional contribution to the activation of the contained neurons of that layer, thus representing their distributed activation contributions. This property is exploited in modern frameworks, such as TensorFlow (Abadi et al., 2015).

**Figure 2:**
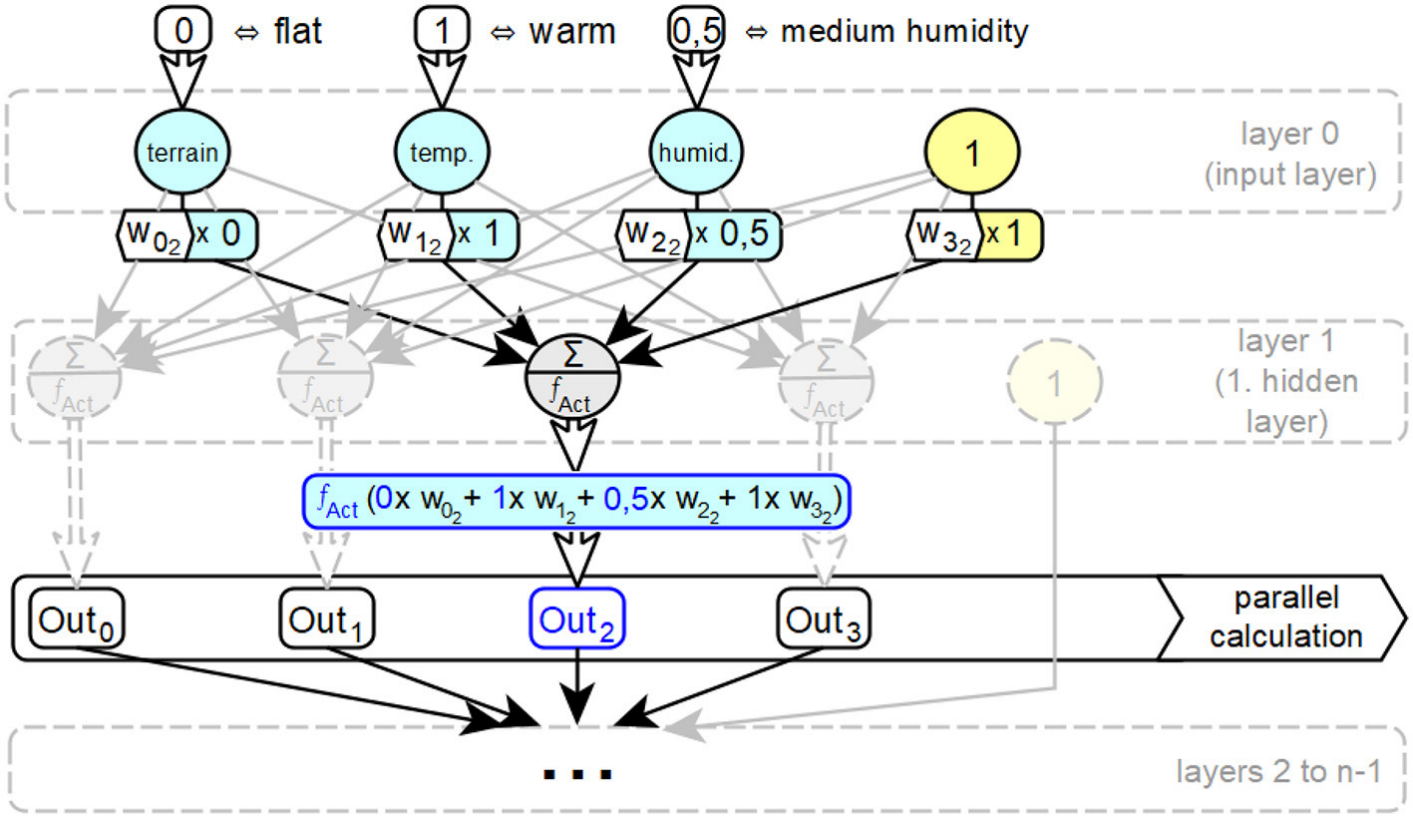
Basic principles of parallel information processing in ANNs with hidden cells.

Figure 2 shows an ANN with *n* layers and the input parameters for altitude, temperature, and humidity. In addition to temperature, input information is given for a flat altitude, coded 0, and for a medium humidity, coded 0.5. In layer 1, the input values of the neurons *i* of layer 0 multiplied by the corresponding connection weight *w*_*i*_*j*__ are processed in parallel by all neurons *j* of this layer as part of the input activation of the neuron *j*. The input activation in the second neuron of layer 1 consists of a *w*_0_2__ component of the representation 0 for the information flat, a *w*_1_2__ component of the representation 1 for the information warm and a *w*_2_2__ component of the representation 0.5 for the information wet. In this step, the connection between the two aspects of artificial neural networks—distributed representation of information and parallel processing of information—becomes clear. Next, this input activation is converted into the output activation of the respective neuron in the layer, in this case the second neuron, by the activation function *f*_*Act*_. These two steps for calculating the output activation *Out*_*j*_ of each neuron *j* of layer 1 are performed in parallel in each neuron *j* in this order. A complete processing cycle through a network for a complete set of inputs is called a *forward pass*.

*Convolutional neural networks* (CNNs) are specialized for image recognition, speech recognition and language processing. The basic idea behind this type of network comes from the realization in Hubel and Wiesel (1962) that the neurons in the visual cortex of animals have only a local field of perception and thus only react to stimuli from a small area of the entire visual field. This later led to the development of a neural model with the *neocognitron* described in Fukushima et al. (1983), which pursued the idea of processing only the activations of spatially adjacent neurons by their successor neurons and thus aggregating individual image sections layer by layer into increasingly extensive representations of partial images. CNNs and the associated properties of their elements can be considered as a special form of feedforward neural networks and can be fully represented by hidden cells, taking into account the relevant specifics (Bach et al., 2015).

In the following, the derivation of *decision trees* as equivalent symbol-based decision models for given feedforward (or convolutional) neural networks will be developed step by step. Other generative artificial neural networks such as Hopfield networks, Boltzmann machines, autoencoders, and long/short term memory networks and their specific elements (van Veen, 2016) are beyond the scope of the derivation to be developed in this work. The research methodology underlying this work is a thorough and systematic investigation of symbol-based and connectionist AI approaches. By providing a prototype implementation, we can demonstrate practical feasibility.

## 3 The derivation of decision trees

Before we start with the derivation, a few terms need to be defined in more detail.

### 3.1 Symbols and combination rules

A *symbol* is a single entity with a defined meaning. Finite sets of these entities can be combined according to defined rules to form new entities, to which a new, defined meaning is then assigned. Thus, there are the following categories of symbols: complete entities, which have a meaning assigned by definition, and composite entities, whose meaning results from their components and their meanings on the one hand, and from the rule for combining these components on the other. Whether an entity is complete or composite depends on the permitted *combination rules*.

When determining the category of a symbol, the permissible set of combination rules must always be taken into account. For example, a letter is a complete symbol if only the combination rules of the respective written language are to be considered. However, if combinations in the sense of pictorial representation are to be included, a letter can be considered a composite symbol consisting of a set of points.

### 3.2 Fillers and roles in feedforward networks

Fillers are assignments, i.e. perceptible representations, for an associated role in a symbol-based overall structure. They are thus a representation of a symbol entity, be it a complete or a composite symbol. Similarly, roles can be considered as the relations or relationships of a symbol entity represented by a filler to all other symbol entities present in a common symbol-based structure. The combinations of fillers and roles are realized in symbol-based information processing by the respective valid combination rules. In this context, the combination rules always refer to the roles, since the fillers are only one, basically freely selectable representation.

This idea was originally presented by Smolensky and Legendre (2006), where symbols consisting of fillers and roles are depicted by numerical activation values forming specific activation patterns. Their combination is then realized as a superpositioning of these vectors in an intermediate vectorial level. The result of this operation can again be regarded as a complete unit, comparable to a single symbol or a symbol-based structure *s*, where $s=\underset{i}{\sum }f_{i}⊗r_{i}$, the sum total of all combinations of fillers *f*_*i*_ and their associated roles *r*_*i*_ contained in *s*. The (*filler*) *f*_*i*_ represents a clearly assignable sign. The structural role *r*_*i*_ describes the relationships of the sign to all other signs in the overall symbol *s*.

**Figure 3:**
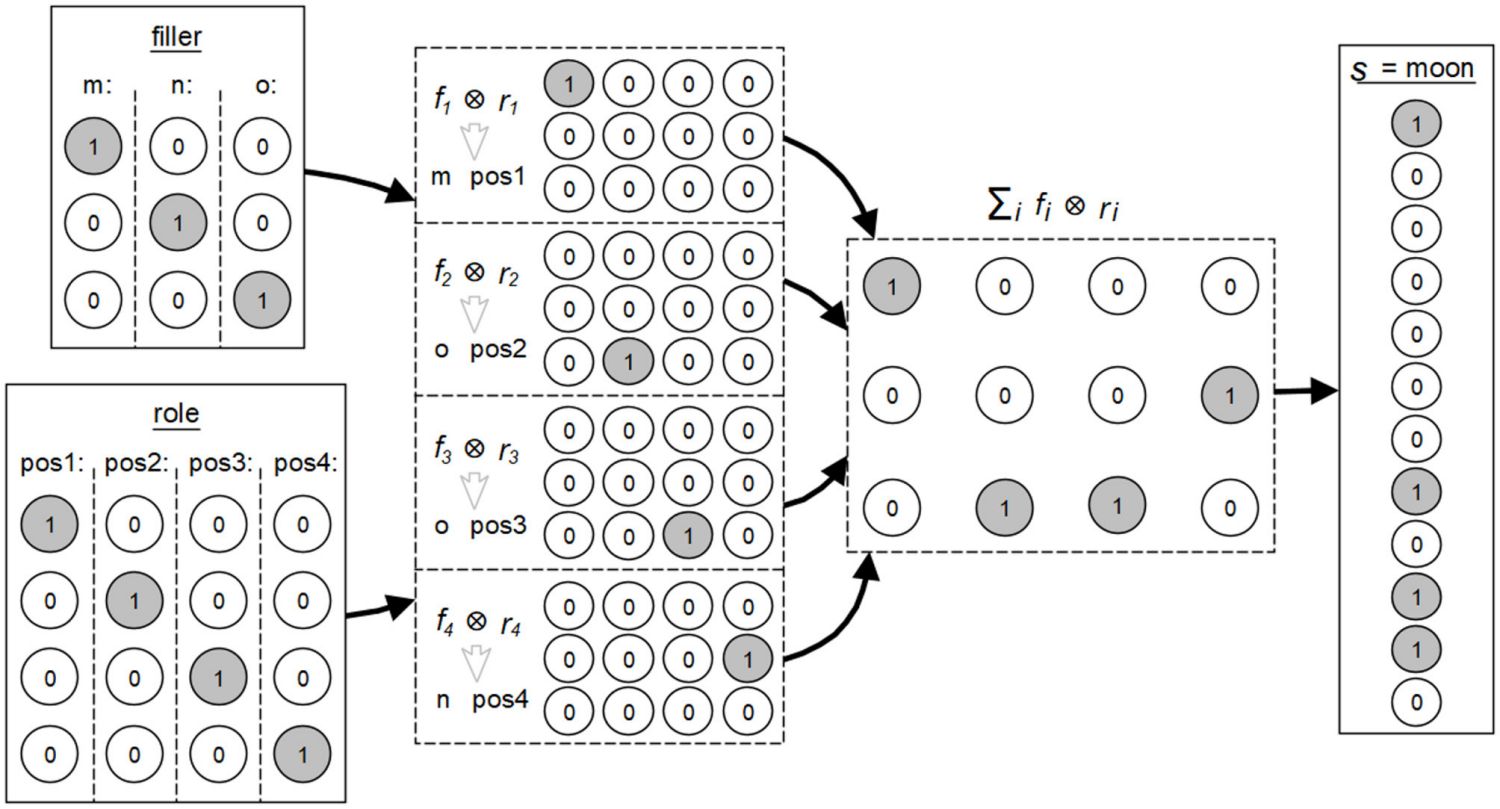
Example of the concept of fillers and the roles in the integrated connectionist/symbolic architecture according to the explanations by Smolensky and Legendre (2006).

A simple example is any word *s* with its specific meaning, formed from all contained letters *f*_*i*_ and their respective positions *r*_*i*_ in that word. Figure 3 illustrates this using the symbol structure associated with the word *moon* according to the concept of the integrated connectionist/symbolic architecture by Smolensky and Legendre (2006). The fillers, here the letters *m, n* and *o*, are each formed by 3-dimensional vectors consisting of binary values. The roles, here the positions at *pos 1, pos 2, pos 3* and *pos 4* in the word, correspond to 4-dimensional vectors of binary values. The summed tensor products of the vectors result in a 3 × 4-dimensional matrix *s*, which in turn can be represented as a 12-dimensional vector. This matrix and this vector are again two representations of the word *moon*.

**Figure 4:**
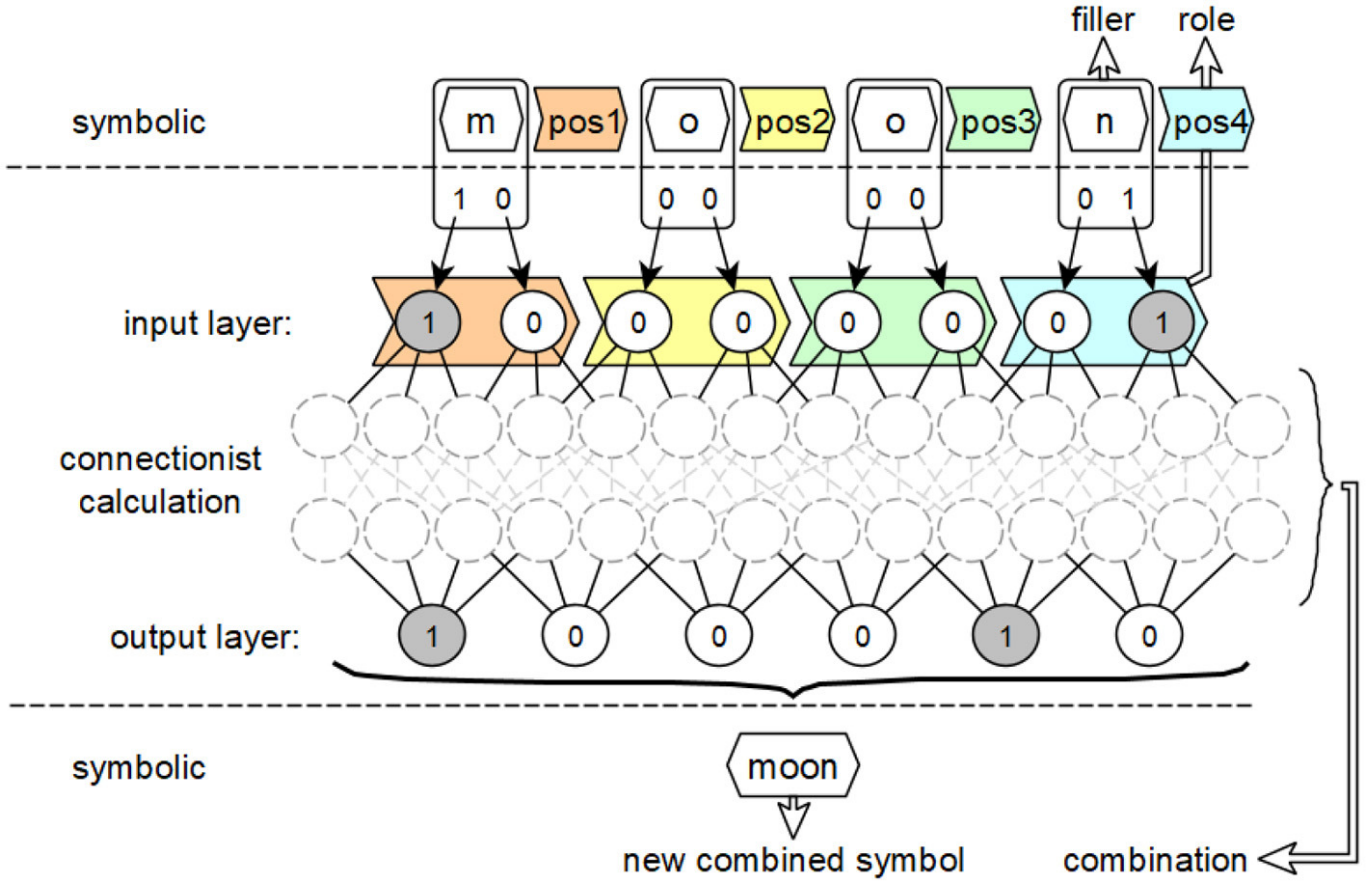
Intended use of the concepts of fillers, roles and combinations of activation patterns from the integrated connectionist/symbolic architecture applied to feedforward neural networks to derive symbol equivalents.

This example also illustrates the connection between the symbol-based, vectorial, and neural levels mentioned above. The transfer of this principle to feedforward neural networks is shown in Figure 4.

The symbolic level, which is adjacent to the input layer in the form of the interpreted input, contains compressed carriers of information meaning, consisting of their filling word, the letter, and their role, the position in the word. The neural layer, realized by the neural network, describes the connectionist derivation of output activations from input activations using activation patterns that implement this derivation. The respective output activations and their associated dependencies on the activation values of the inputs describe how the new overall symbol is formed from the fillers and roles of the individual symbols of the input.

While fillers and roles for the generation of symbols can be determined from the inputs of a feedforward neural network, if necessary by inverting the preprocessing of these inputs, the combination of these fillers and roles is found in the structure of the respective networks. A single combination is realized by the incoming weighted connections *w*_*j*_ and the corresponding transmitted activation values *v*_*j*_ of a single neuron in a subsequent layer.

This layer follows each layer in the examined feedforward network for whose neurons valid symbol equivalents with associated fillers and roles have already been determined. The input value for each neuron is determined by the sum $\underset{i}{\sum }v_{i}\times w_{i}$. This sum represents the function, also called pattern or rule, by which the composite symbol associated with the neuron in question is determined in relation to its predecessor neurons. All symbols with their associated fillers and roles are potentially considered for those combinations that are associated with a predecessor neuron that can be reached via an incoming connection *j*. If the product *v*_*j*_×*w*_*j*_ satisfies


(1) $$\begin{array}{l} \frac{v_{j}\times w_{j}}{\sum_{i}v_{i}\times w_{i}}\geq \theta \;\text{and }v_{j}\times w_{j}\geq \varepsilon , \end{array}$$

where θ∈(0, 1) is a relative relevance threshold applied layerwise and ε>0 is an absolute activation cutoff, then the activation of neuron *j* is considered significant for $f_{\text{Act}}\left(\underset{i}{\sum }v_{i}\times w_{i}\right)$ with *j*∈{*i*}. After the combination, the resulting symbol for the target neuron consists of the set of all tuples (filler_*j*_, role_*j*_) whose associated product *v*_*j*_×*w*_*j*_ is relevant for $f_{\text{Act}}\left(\underset{i}{\sum }v_{i}\times w_{i}\right)$ with *j*∈{*i*}. The output activation $f_{\text{Act}}\left(\underset{i}{\sum }v_{i}\times w_{i}\right)$ calculated in the target neuron must be relevant for the input activations of the successor neurons, so that the resulting symbol is considered for the combination with the linked symbol of the respective successor neuron.

Since it is possible with the steps described above to generate both fillers and roles, as well as their combinations, from individual elements of the complete computation of a decision in a feedforward network with associated inputs and outputs, these concepts can be used in the further course of this work as bridging elements between feedforward networks and decision models that use symbols. The idea here is to first determine the fillers and roles for the calculation of a decision and then the respective valid linked sets of tuples *T* = (filler, role) and thus to generate the necessary symbol equivalents for a symbol-based decision model equivalent to the examined feedforward network.

### 3.3 Decision trees and feedforward neural networks

To derive an equivalent decision tree from a given neural network, the structure of the underlying graph must be created in an additional step. This graph connects the later symbol equivalents. However, there are several slightly different representations of decision trees in Géron (2022), Millington and Funge (2009), Rokach and Maimon (2005), and Seidel and Borghoff (2018).

A decision tree is a directed graph. It starts in exactly one node, ends in decisions *D* and consists of nodes *N* and edges *E*. The nodes *n*∈*N* are described as tuples *n* = (*s, t*) where *s* is a defined information state *s* = {*i*_0_, …, *i*_*k*−1_} consisting of *k* individual items of information *i*_*j*_ with *j*∈{0, …, *k*−1}. Each individual item of information *i*_*j*_ is assigned a set of *m* possible values *b*_*i*__*j*__*r*___∈{*i*_*j*_0__, …, *i*_*j*_*m*_−1_} with *r*∈{0, …, *m*−1}. The assignments are represented by symbols. *t* is a test on at least one piece of individual information *i*_*j*_, so that *s*×*t*×*b*_*i*__*j*__*r*___→*e*_*r*_ applies with edge *e*_*r*_∈*E* = {*e*_0_, …, *e*_*m*−1_} and *m*= number of outgoing edges. The edges *e* are described by their connection destination *g*. Here, *g*∈(*N*∪*D*). Each edge is linked to the validity / occurrence of an assigned allocation. The decisions *d*∈*D* are described by their specific output value.

**Figure 5:**
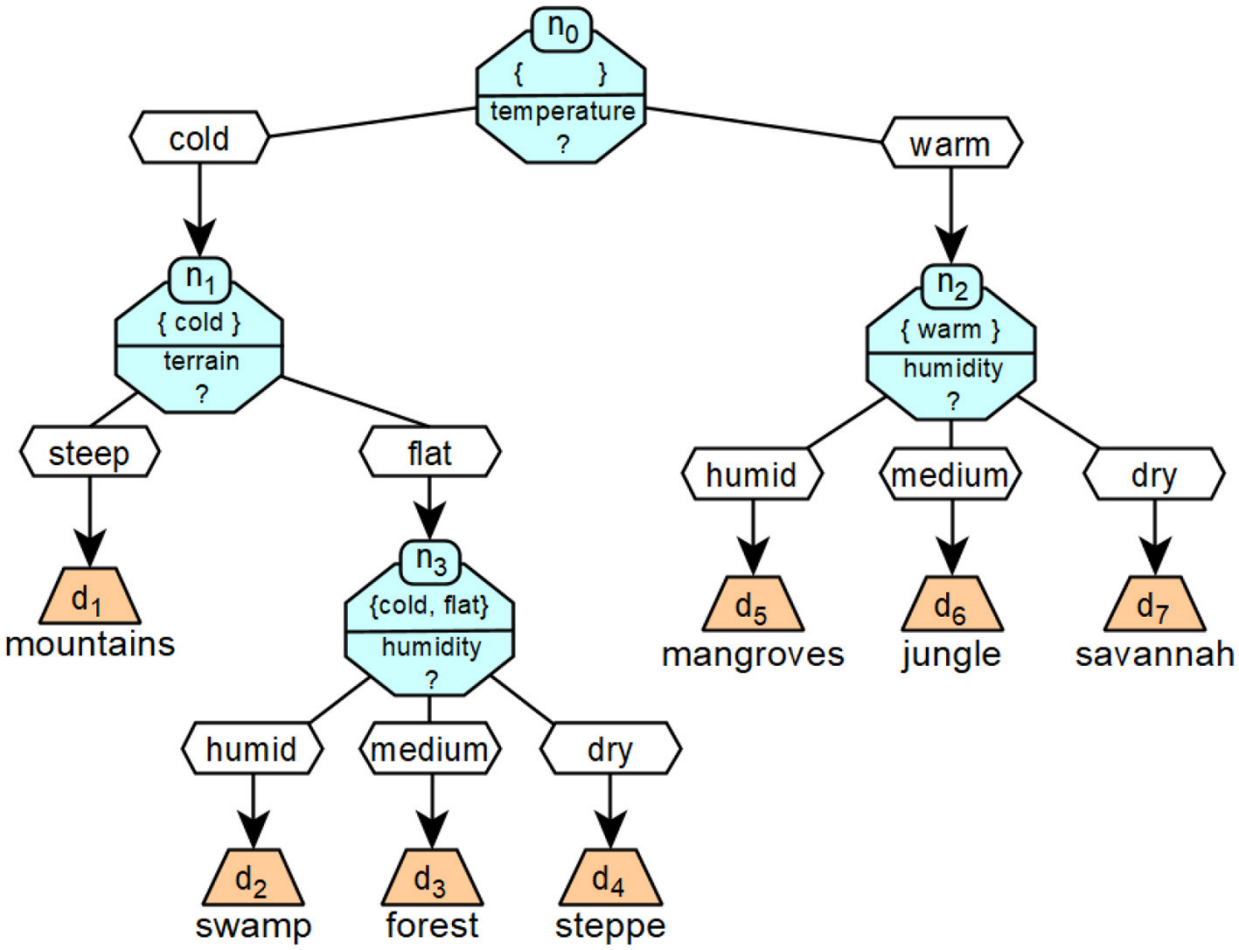
A concrete realization of a decision tree that corresponds to the formalized example introduced in Figure 6.

Figure 5 shows an example of a specific decision tree. The decision tree classifies a given landscape into the seven categories mountain, swamp, forest, steppe, mangrove, jungle and savannah, which are described by the respective decisions *d*. The classification is based on information *i*_*j*_ about the qualitative temperature (*i*_0_), altitude (*i*_1_) and humidity (*i*_2_) of the landscape. The possible values of *b*_*i*_0__ are cool and warm, *b*_*i*_1__ flat and steep and *b*_*i*_2__ wet, medium and dry.

**Figure 6:**
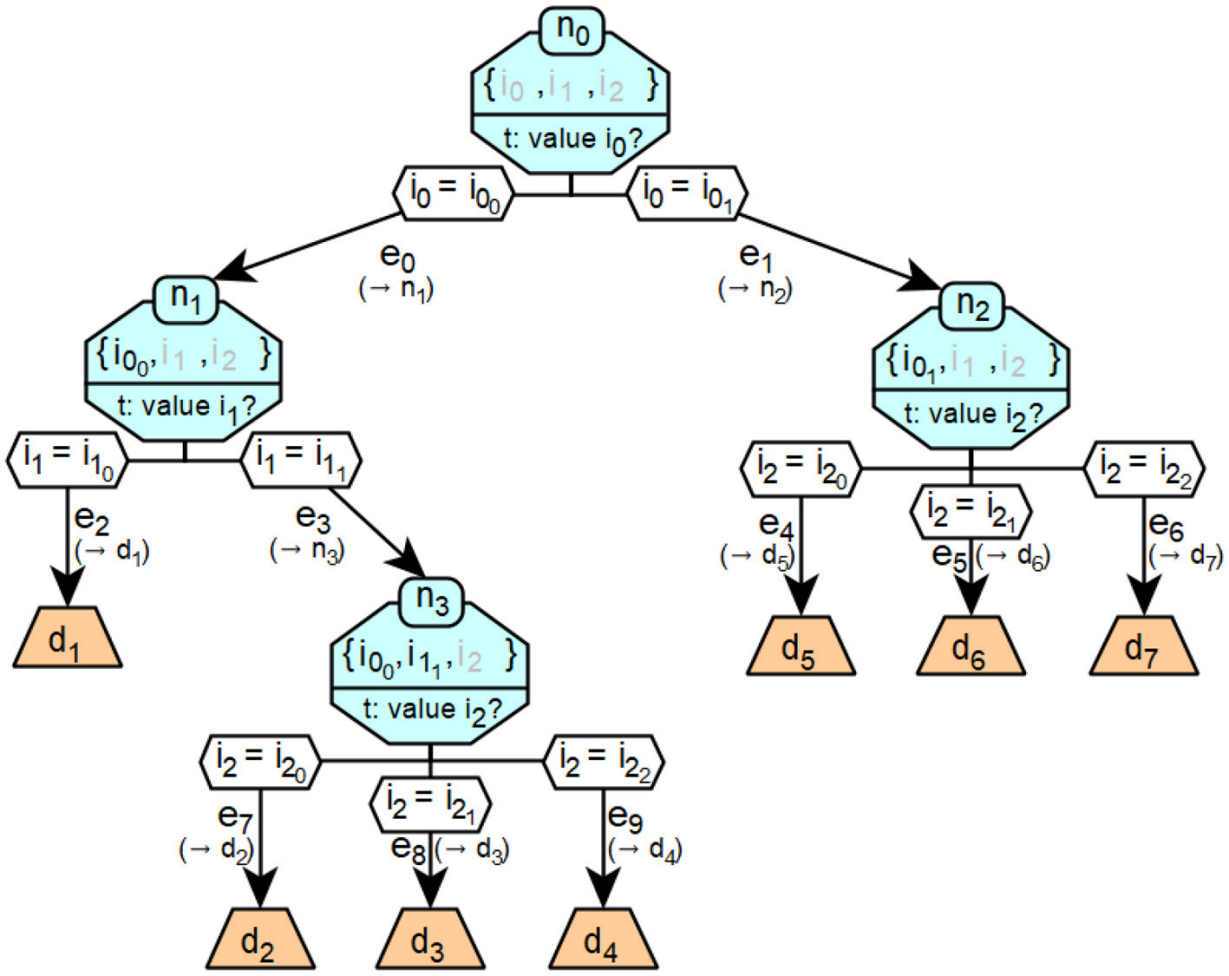
Example of a formalized decision tree.

Figure 6 shows a more formalized version of the decision tree from Figure 5.

### 3.4 Identification of symbols and their relationships to define the structures to be derived

In the nodes *N* of a decision tree, a test *t* contained in the node is assigned a certain possible assignment *b*_*i*__*j*__*r*___ to a single piece of information *i*_*j*_ belonging to that test, or to a certain set of such single pieces of information. The assigned mappings *b*_*i*__*j*__*r*___ follow the principle of fillers associated with their respective roles. So they are assignments with symbols. The information states {*i*_0_, …, *i*_*k*−1_}, over which the tests *t* are performed, correspond to the principle of possible roles to be assumed. Together with an associated filler from an assignment, they form a complete symbol. A defined connecting edge *e*_*r*_ is always connected to this symbol {*i*_0_, …, *i*_*k*−1_} × *b*_*i*__*j*__*r*___ and thus the following node *n* or the corresponding decision *d* of this edge. The symbols {*i*_0_, …, *i*_*k*−1_} × *b*_*i*__*j*__*r*___ can be found in decision trees in the edges *e* when they appear for the first time in response to a test *t*, as well as in the information states *s* of all nodes *n* following the said *e*.

The structure of a decision tree can thus be represented by the symbol components mentioned. Deriving these components from a neural network is a prerequisite for generating an equivalent decision tree for a set of decisions computed by that neural network. These components can be used to generate both the structure of the tree and the required symbolism.

Therefore, the components needed to determine how to structure a decision tree are as follows.

- the test *t* checked in the respective node over a defined set of individual pieces of information and the associated known information states {*i*_0_, …, *i*_*k*−1_}. The information states correspond to already existing/valid combinations of fillers and roles. The tests are used to determine valid fillers for roles that have not yet been filled.
- the possible combinations of assignments *b*_*i*__*j*__*r*___ after the respective node, which represent a valid answer to the test of the node. These correspond to the existing/valid combinations of fillers and roles after the test.
- the edges *e*_*r*_ associated with the valid combinations of assignments to the respective subsequent nodes or subsequent decisions.

In order to determine these components, we will now take another look at the elements offered by the basic form of neural networks.

The edges *e*_*i*_*j*__ run in a directed manner between an input neuron *i* and a target neuron *j* and each has a weight *w*_*i*_*j*__. This weight is a numeric value that is multiplied by all activation values *v*_*i*_ of the input neuron *i* of the edge that are passed along this edge. Input neurons a have an input value *Inp* and a set of *n* output edges {*e*_*a*_0__, …, *e*_*a*_*n*−1__}. Hidden neurons b have a set of *m* input edges {*e*_0_*b*__, …, *e*_(*m*−1)_*b*__} and a set of *n* output edges {*e*_*b*_0__, …, *e*_*b*_*n*−1__}. Furthermore, each of these neurons has an activation function *f*_Act_ and an input function, which usually corresponds to the sum function. Output neurons c have a set of *m* input edges {*e*_0_*c*__, …, *e*_(*m*−1)_*c*__} and an activation function *f*_Act_ as well as an input function, which usually corresponds to the sum function. In addition, each output neuron has an output value *Out*.

When a neural network is created, it is determined which input neurons encode a particular piece of information. The coded information is therefore always known, as can be seen from Goodfellow et al. (2016) and Géron (2022), among others. This coded information corresponds to the individual information *i*_*j*_ from decision trees. This also allows to derive the tests *t*, i.e. the information *i*_*j*_ on which these tests have to be performed. The assignment of the tests to a node and the associated information states is not yet possible. The respective combinations of input values *Inp* correspond to the specific assignments *b*_*i*__*j*__*r*___ of the information *i*_*j*_ of a decision tree, which apply to a defined, associated decision *d*. These combinations thus also realize the concept of fillers according to Smolensky and Legendre (2006). The coding of the possible decisions of a neural network by the output values *Out* of the output neurons is also known, since this is also determined when the neural network is created.

Each output vector of a neural network is associated with a specific decision, which is equivalent to the decisions *e* of the decision trees. The individual inputs for the coded information, which correspond to specific assignments *b*_*i*__*j*__*r*___, and the resulting output of the neural network, which corresponds to a single decision *d*, are each assigned to a decision process of a neural network that has been run through. Their equivalents can therefore be regarded as individual elements of a specific path through a decision tree, hereafter referred to as a decision path, which leads to exactly the decision *d* that corresponds to the output of the neural network for the decision made.

**Figure 7:**
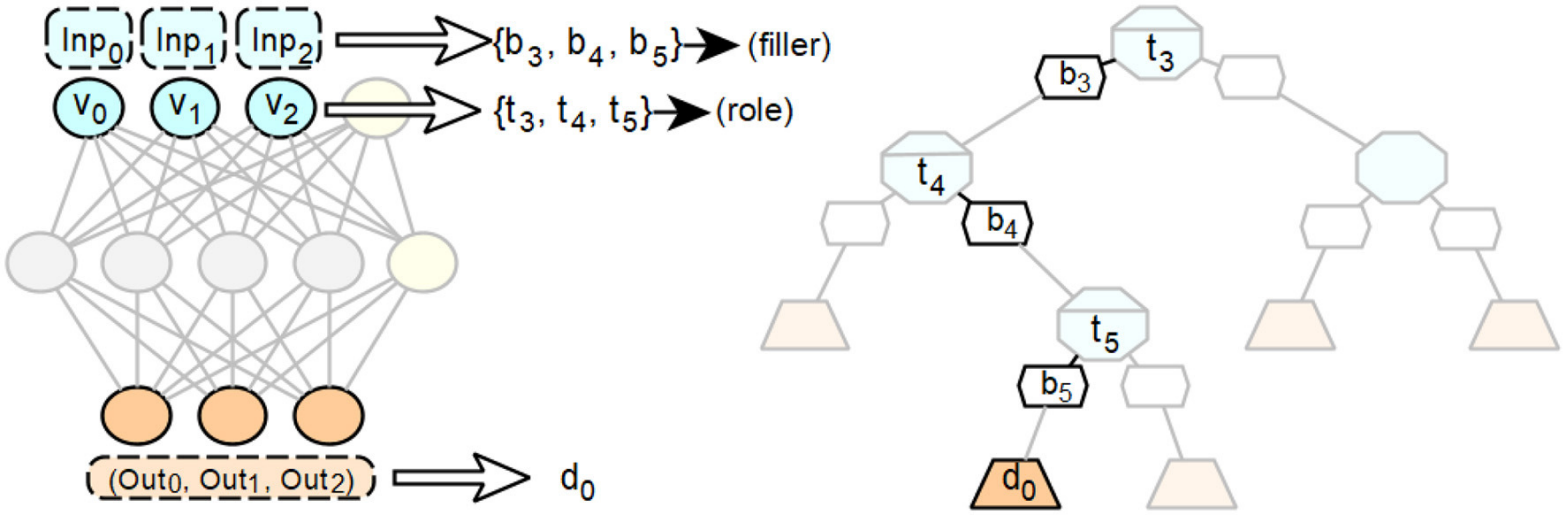
Exemplary representation of the elements of a single path of a decision tree that can be derived from a single run of a given feedforward neural network.

See Figure 7 for an example.

In order to be able to make statements about the arrangement of the pairs from a test *t* and the associated subsequent assignment *b*_*i*__*j*__*r*___, it is first clarified which properties are associated with the position of such a pair at a certain point in a decision path. Then, the internal structure of the neural network used for the decision is taken into account in order to determine subgraphs that allow conclusions to be drawn about the desired properties. In doing so, the peculiarities of the processing of individual pieces of information *i*_*j*_ and their values *b*_*i*__*j*__*r*___ to generate a decision in neural networks must be taken into account. In particular, the distributed representation of information must be considered. The properties to be considered for the position of a pair (*t, b*_*i*__*j*__*r*___) are:

- once a pair (*t, b*_*i*__*j*__*r*___) has an assigned position in a decision tree, the assignment *b*_*i*__*j*__*r*___ must be the valid assignment for the associated information *i*_*j*_, which applies to all subsequent decisions *d* after this position. In particular, this follows from the approach *s* × *t* × *b*_*i*__*j*__*r*___ → *e*_*r*_ for determining the successor edges, since the decisions *d* are at the end of an associated cascade of edges.
- there is a direct dependency of the decisions *d* on the preceding valid assignments *b*_*i*__*j*__*r*___.
- there is a direct dependency among the assignments *b*_*i*__*j*__*r*___ of a single piece of information *i*_*j*_. Once a position in a decision tree has been determined for an assignment of a piece of information, no other possible assignment for the same information can follow after this position in the respective decision path.

The assignments of the input values *Inp* are not recorded and processed in a single place in the hidden layers that follow the input layer, but are distributed in different places. This means that they are distributed and proportionally included in the determination of the overall decision. In principle, each neuron corresponds to a function that converts an input vector into a scalar. The input vector in turn contains the scalars of the previous layer as components, which for the neurons of the first hidden layer are the concrete numerical values of the respective *Inp* values. Thus, with increasing depth, the hidden layers contain more and more partial representations or assignments of an *Inp* value until the final decision is made.

The output *Out* of a neuron is $Out=f_{Act}\left(\underset{i}{\sum }v_{i}\times w_{i}\right)$ with *v*_*i*_ = input value *i* and *w*_*i*_ = weighting factor for input value *i*. The value *Out*, which is either an intermediate or a final result of the decision calculation, always depends on the sum of the total input and thus on the complete input vector over all weighted input values (proportional occupancy). The basis for this is again the distributed representation of information in artificial neural networks combined with parallel processing of this information. In multilayer neural networks, this leads to concatenated or cascaded combinations of the original input values across the successive layers of such a network. For a neural network with an output layer *n*, this corresponds to the form $Out=f_{Act_{n}}\left(\underset{i}{\sum }\left(f_{Act_{n-1}}\left(\underset{j}{\sum }\left(...\right)\times w_{j}\right)\right)\times w_{i}\right)$.

**Figure 8:**
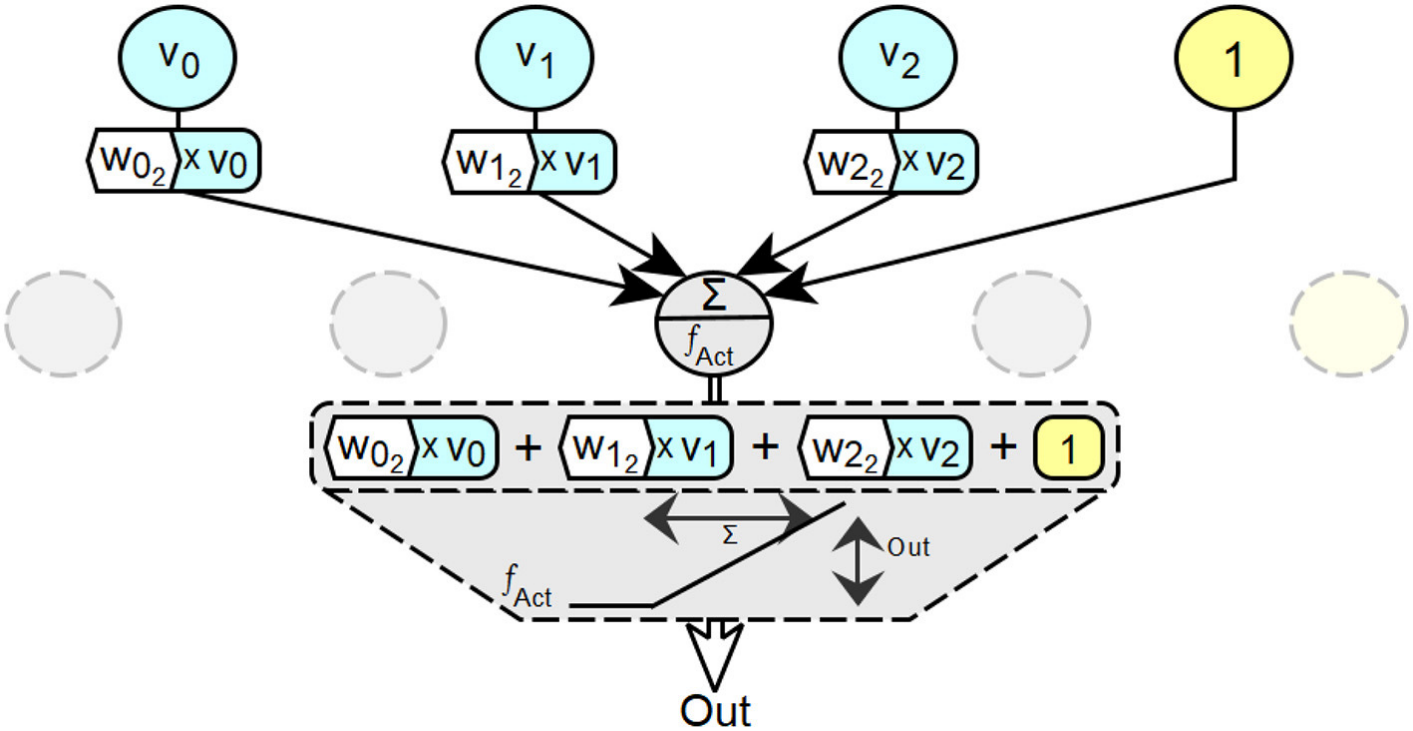
Exemplary visualization of the principle of dependence of individual occupancy values on valid partial occupancies at specific locations (neurons) in neural networks.

The validity of a partial input value at a specific location in a neural network as part of the calculation of a desired output always depends on the simultaneous validity of all other partial input values at that specific location which, in combination with the input value under consideration, lead to the desired output. This is illustrated in Figure 8.

The properties of feedforward neural networks just described are essential for the process of input processing to compute a decision and to determine the symbol components fillers, roles and their combination in the structure of feedforward networks and assign them to the respective neurons. First, we have to determine which proportional distribution of the assignments of the input values in a single processing step contributes to the calculation of the output vector in the output layer and thus to the generation of the resulting decision. The relevant proportions of the distributed representation are passed from the penultimate layer to the output layer. Therefore, the neurons of the penultimate layer of the considered network that send a value of sufficient magnitude to the output layer to make a relevant contribution to the generation of the overall output should be examined.

For each of these neurons *j* of the penultimate layer, it must be determined for each input neuron whether this value makes a sufficiently large numerical contribution, analogous to Equation 1. The following steps are carried out to capture relevant distributed representations of input configurations in a decision path for the computed decision *d*.

1. if a neuron *j* has no connection to a neuron *k* in the output layer such that^1^

(2) $$\begin{array}{l} \frac{v_{jk}\times w_{jk}}{\sum_{l}v_{jl}\times w_{jl}}\geq \theta \;\text{and }v_{jk}\times w_{jk}\geq \varepsilon . \end{array}$$

then neuron *j* is excluded from the decision path since it fails to meet the relevance conditions.
2. if an input neuron's assignment is not relevant enough to activate neuron *j*'s output in the above definition because there are no sufficiently weighted connections to neuron *j*, then this input neuron's assignment is not considered in neuron *j*'s decision path in a decision tree.
3. otherwise, the corresponding input assignment for the respective neuron *j* is taken into account in the considered path of the decision tree.

**Figure 9:**
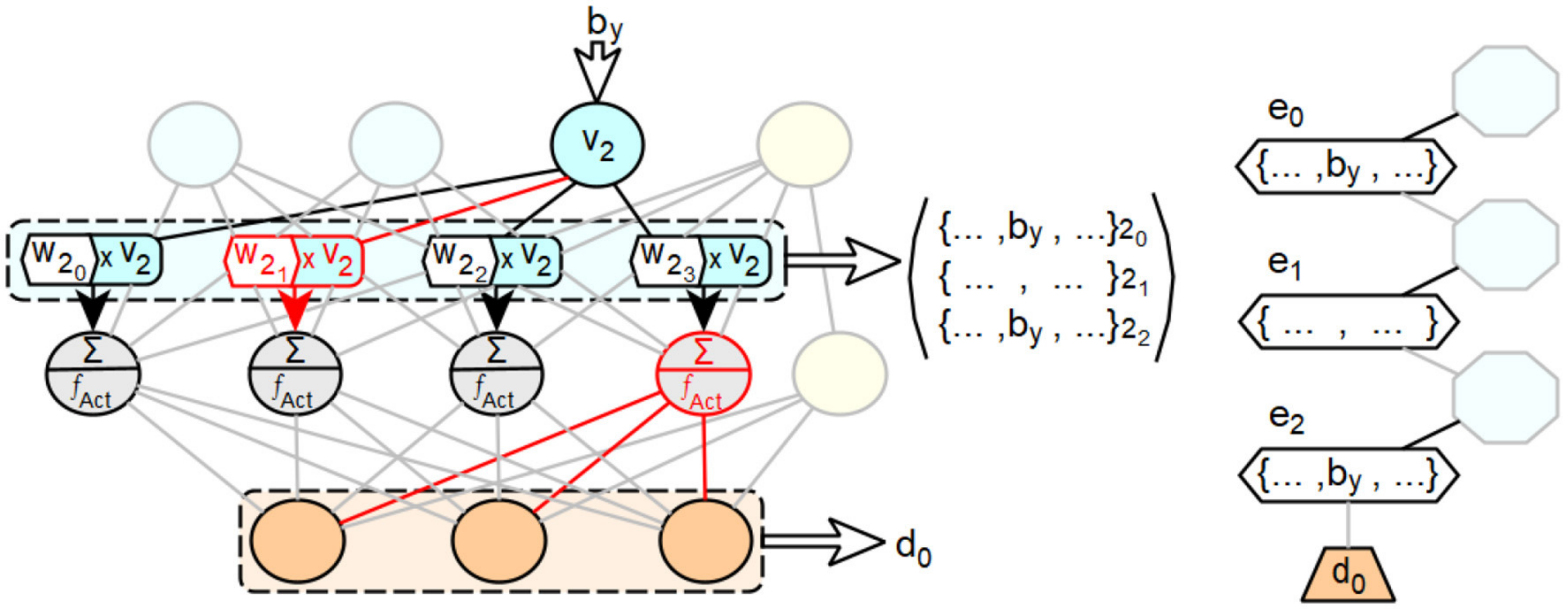
Exemplary illustration of the concept of integrating distributed representation and processing of feedforward neural networks into extended decision trees.

The arrangement of the neurons of the penultimate layer is arbitrary, since they are simultaneously considered as neurons of the same layer in the processing of the feedforward neural network. A visualized example of the described procedure is shown in Figure 9. It shows a small feedforward neural network with an input layer consisting of three neurons and a bias neuron, a hidden layer with four neurons and a bias neuron, and an output layer consisting of three neurons. The last neuron of the penultimate layer has no connections through which a relevant activation value for the current input to the network is passed to a neuron of the output layer, resulting in the output *d*_0_. Consequently, the set of input configurations that produce an activation in this neuron is not considered in the edges of the decision path for *d*_0_. The other three neurons of the penultimate layer are considered. However, the second neuron of the penultimate layer has no connection through which it can receive an activation value that is relevant for the current input and that includes the value *b*_*v*_ of the third input neuron. Therefore, *b*_*y*_ is not considered as an allocation for the edge derived from the second neuron.

### 3.5 Extension of decision paths for use in deep feedforward neural networks

Modern feedforward neural networks typically have multiple hidden layers. These layers, like the penultimate layer of a feedforward neural network, represent parts of the processing of the network's input assignments into the corresponding network output. Therefore, to fully account for the aforementioned processing by a derived decision path, all hidden layers of the considered network must be considered in that path.

These processing steps are represented by the subnetworks of the considered network, which start with all input neurons and end with the individual neurons assigned to the edges *e*. From these subnetworks decision paths can be derived, which replace exactly the edge *e* in the decision path already derived from the whole network, which was derived from the target neuron of the considered subnetwork. This procedure can now be repeated layer by layer, and in these layers neuron by neuron, until each hidden neuron with a relevant activation in the analyzed feedforward neural network is represented by at least one associated edge in the derived decision path.

**Figure 10:**
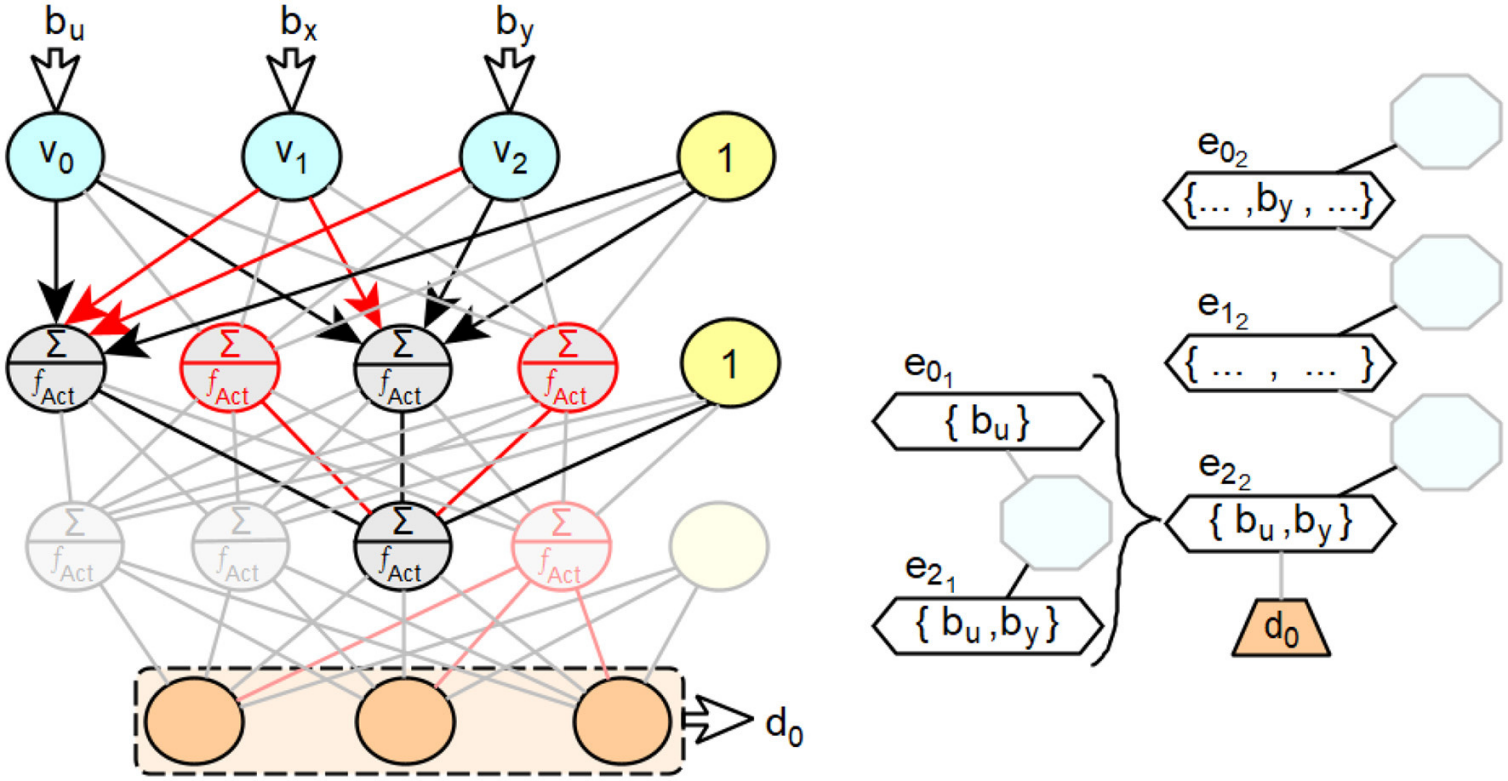
Exemplary illustration of the expansion of decision paths in extended decision trees for feedforward neural networks with several intermediate layers.

Using Figure 10 as an example, we will describe the procedure for a feedforward neural network with two hidden layers. For the third neuron of this layer, the relevant processing steps of the second layer are to be taken into account. In the example shown, the said third neuron of the second layer receives the sufficiently large weighted activations *v*_0_×*w*_0_2__ from the first neuron and *v*_2_×*w*_2_2__ from the third neuron of the second layer.

Accordingly, the new edges *e*_0_1__ for the first neuron and *e*_2_1__ for the third neuron are created in the new subpath associated with the edge *e*_2_2__. The absolute values of the weighted activations of the second and fourth neurons of the second layer are too small and thus insignificant for the overall input activation of the third neuron of layer three. For the neuron assigned to the edge *e*_0_1__, only the weighted activation of the input neuron *v*_0_ is large enough in absolute value to be relevant to its overall input activation. Therefore, only the input weight *b*_*u*_ of *v*_0_ is assigned to the edge *e*_0_1__. For the neuron associated with the edge *e*_2_1__, the weighted activations of *v*_0_ and *v*_2_ are relevant due to their magnitude. Therefore, the input weight *b*_*u*_ of *b*_*y*_ is assigned to *e*_2_1__. In the following, a procedure for deriving decision paths from feedforward neural networks with more than one hidden layer is described, based on the statements made so far.

We now have all the ingredients for the final derivation procedure. At the beginning, the output mapping and the associated decision are computed for the given vector of input mappings. At the end of this calculation, the respective output activations *v*_*j*_ generated by the given input vector are also computed for all neurons *j* of the hidden layers and the output layer.

Then, for a feedforward neural network with 1 to *n* hidden layers, the following steps are to be carried out for each hidden layer *s* with *s*∈{1, …, *n*} in descending order starting from layer *n* for all *m* neurons of the respective layer *s*. Here, the neuron *j* with *j*∈{0, …, *m*−1} is the neuron of layer *s* currently being considered:

1. if the successor layer of the neuron *j* is the output layer, then it is necessary to check if there is a connection to a neuron *k* of the successor layer through which a sufficiently large, w.r.t Equation 2, weighted value *v*_*j*_*s*__×*w*_*j*_*k*__, which is significant for the calculated output activation, is propagated. If this is the case, the neuron *j* of layer *s* is considered with a new associated edge *e*_*j*_*s*__ at the end of the decision path to be derived.
2. if the successor layer of the neuron *j* is a hidden layer, it is necessary to check whether there is a connection to a neuron *k* of the successor layer, which is already considered with an edge *e*_*k*_*s*+1__ in the decision path to be derived, and whether a sufficiently large weighted value *v*_*j*_*s*__×*w*_*j*_*k*__ is transmitted over this connection, which is significant for the calculated activation of *k*. If this is the case, the neuron *j* of layer *s* is considered with a new associated edge *e*_*j*_*s*__ at the end of the partial path to be derived, which is assigned to the already existing edge *e*_*k*_*s*+1__.

Once the described procedure has been completed, a complete derived decision path with all considered decision levels, i.e. layers of the original feedforward neural network, is available for the considered decision. A single edge *e* of this path is either directly assigned a set of relevant input configurations or a subpath contained in it. If *e* is assigned a subpath, the associated set of relevant input configurations is formed from the union of all sets of relevant input configurations of the edges of the assigned subpath.

## 4 The prototype

The prototype was created using JDK 12.0.1, with the graphical display based on JavaFX. Typically, TensorFlow creates a computational graph that allows a just-in-time compiler to optimize its computations. Furthermore, this graph can be stored in a portable format and then run in other environments. However, this is not a useful approach for our prototype, since access to the internal architecture and operations of the model is not sufficient for the implementation of the method developed in this work. The tf.keras module, on the other hand, provides a high-level API that makes it relatively easy to develop models. In the context of the prototype, however, it is particularly important that models trained with Keras can be saved and stored in external files in the .h5-format. This means that information about these models can basically be viewed outside of TensorFlow, which is essential for using the aforementioned models for demonstration purposes in the prototype.

As described by the HDF Group by HDFGroup (2006), the Hierarchical Data Format 5, or HDF5 for short, is a concept for storing and managing complex and memory-intensive data. It consists of a data model, a file format, and libraries and applications for using the format in combination with the associated data model. The .h5-files, in which the information about the trained Keras models is stored externally, belong to this file format. The model architecture, which contains information about the individual neuron layers and their parameters, as well as the respective connections between these layers are stored in the .h5-file as model_config in the form of a JSON string. The values of the individual weights for each connection are stored within the .h5-file as lists grouped by the names of the associated layers.

JavaScript Object Notation, JSON (JSON.org, 2017), is a lightweight data exchange format in the form of a text format based on a subset of the JavaScript programming language, standard ECMA-262, third edition of December 1999, and defined in standard ECMA-404. The advantages of JSON are its ease of creation and readability for humans—which is central to us—and its ease of creation and parsing by machines. JSON uses ordered lists and name-value pairs to create a structure and to store objects, arrays, values, strings, and numbers in that structure. The MapperService for reading information from .h5-files relies on the independently developed jHDF library by Mudd (2015).

### 4.1 The layers

**Figure 11:**
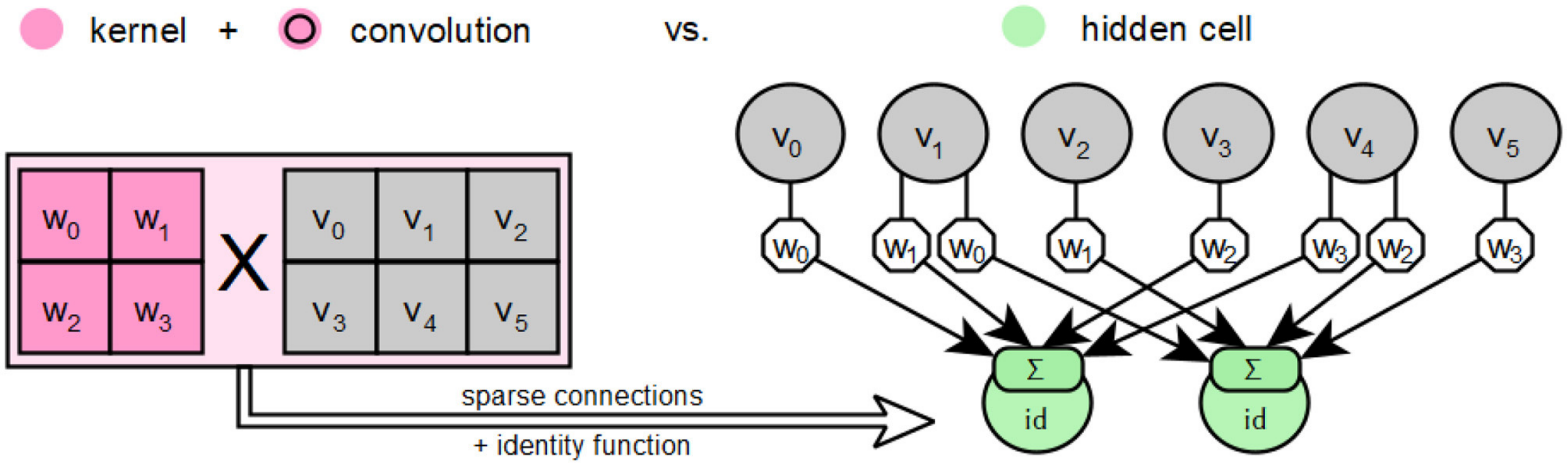
Description of the special features of a convolution using a kernel compared to hidden cells based on the descriptions by Goodfellow et al. (2016).

In Keras, the *input layer* is defined as an object of type Input, as described by Chollet (2015) under *Layers API / Core layers / Input object*. This object represents the input function of an artificial neural network. This layer provides the values of the input vectors to the neural network in a defined form to be passed through the weighted input connections of the following layer. Keras has no explicit output layers. Instead, the other available layer types serve as *output layer* when instantiated as the last layer in a neural network. However, the last layer of a neural network is usually used in conjunction with a SoftMax function as the activation function. *Feedforward layers* are implemented in TensorFlow with Keras using the Dense class, which already has a weight matrix for the input connections of its neurons via the kernel attribute. This weight matrix corresponds to a complete connection of the neurons to the previous layer, hence the name Dense, and can be used in the prototype to map the layer directly back to its feedforward form. The basic *convolution layers* are provided in Keras with the classes Conv1D, Conv2D and Conv3D, which are based on the superclass Conv, whose convolution operation _convolutional_op uses the TensorFlow method convolutional_op. on_v2 from the tf.nn_ops package. This method is offered for use via the tf.nn package with the interface name convolution. Therefore, for objects of the mentioned classes, a convolution kernel is stored in the .h5-file under filters and not an already completed weight matrix as it is the case for objects of the Dense class. It is also explained for the tf.nn module that the respective calculations of the weighted connections using the convolutional kernel depend on the applied zero padding, which is determined by the padding parameter (Abadi et al., 2015). For further processing by the prototype, the convolutional layers are first converted from their special form into an equivalent feedforward form. This is always possible, since a convolutional layer is always functionally equivalent to a sparsely connected feedforward layer, as shown schematically in Figure 11.

**Figure 12:**
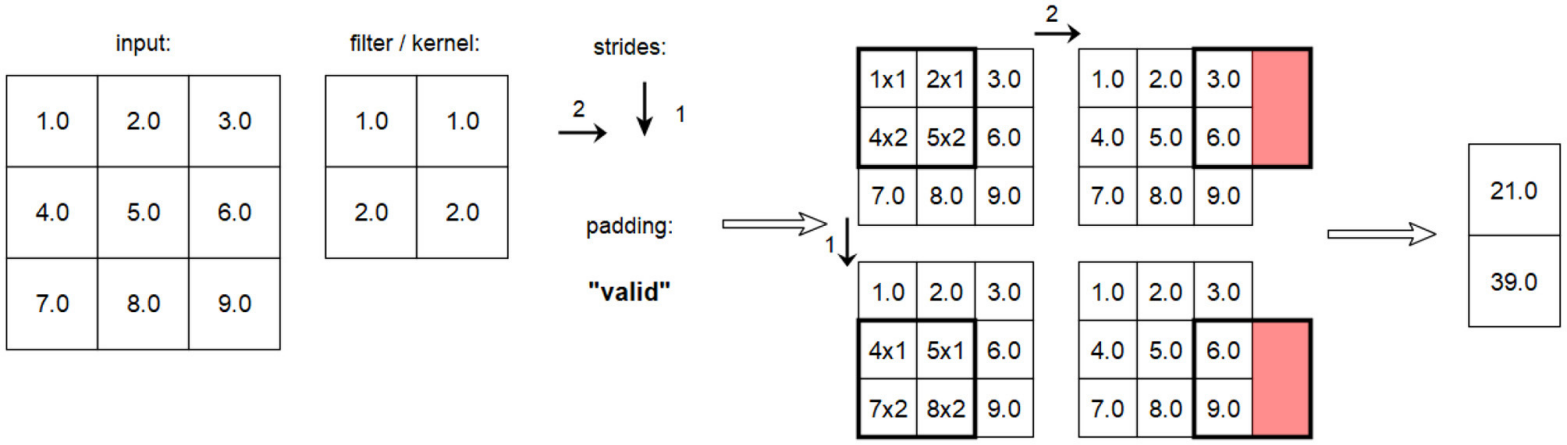
Example of a Conv2D layer processing a 2D input with given strides [2,1] and valid padding.

For a correct implementation, the additional attributes of the convolution, i.e., the padding and the steps used in each dimension, must also be included. An example of such a complete convolution according to TensorFlow is shown in Figure 12.

**Figure 13:**
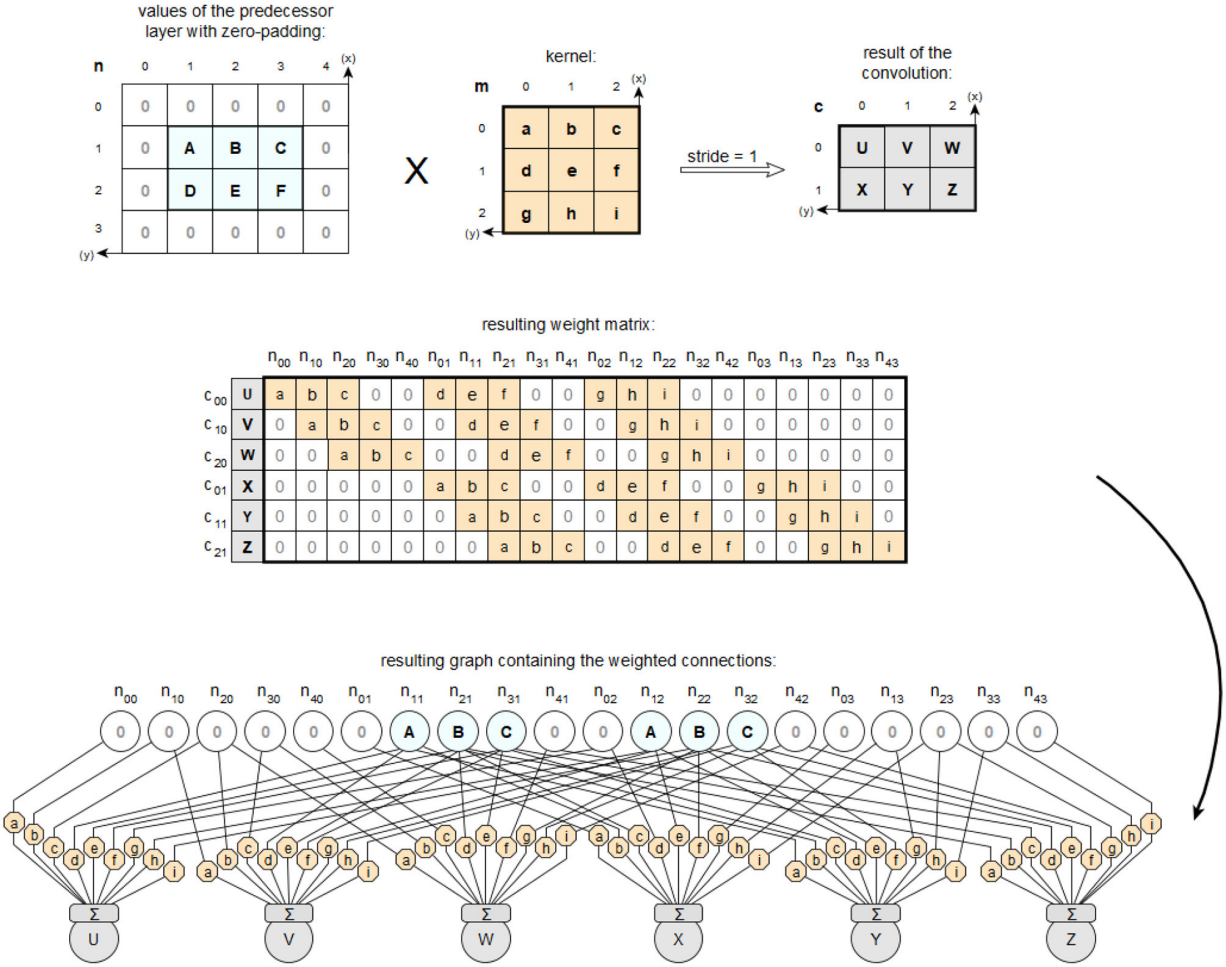
Example of the transfer of a 2D convolution into the filter of a feedforward layer using same padding.

To convert the convolution kernel into a feedforward convolution layer, the convolution kernel is created as a separate object with stored functionality. The prototype can currently handle 1D, 2D, and 3D convolutions. Then the Neuron objects for the layer's NeuralLayer object are created by the corresponding kernel belonging to the appropriate subclass of CNNKernel. After the setNeurons method of the MapperService has been fully processed for the NeuralLayer object of a layer, all filters and their Neuron objects with associated input weights are available in this object, mapping the feedforward form of the respective layer with all required functionalities. A schematic representation of the components involved in this procedure is shown in Figure 13, using a 2-dimensional example convolution, here with equal padding.

### 4.2 The pooling

In Keras, *pooling* is implemented using classes for MaxPooling, AveragePooling, Global, MaxPooling and GlobalAveragePooling, where layer types for 1-dimensional, 2-dimensional and 3-dimensional input are available for these pooling types. Pooling is performed separately for each filter in a layer. For the prototype, only max-pooling and thus the classes MaxPooling1D, MaxPooling2D and MaxPooling3D are considered. Since the spatial processing of pooling is analogous to that of convolution, it is again possible to specify a padding parameter.

**Figure 14:**
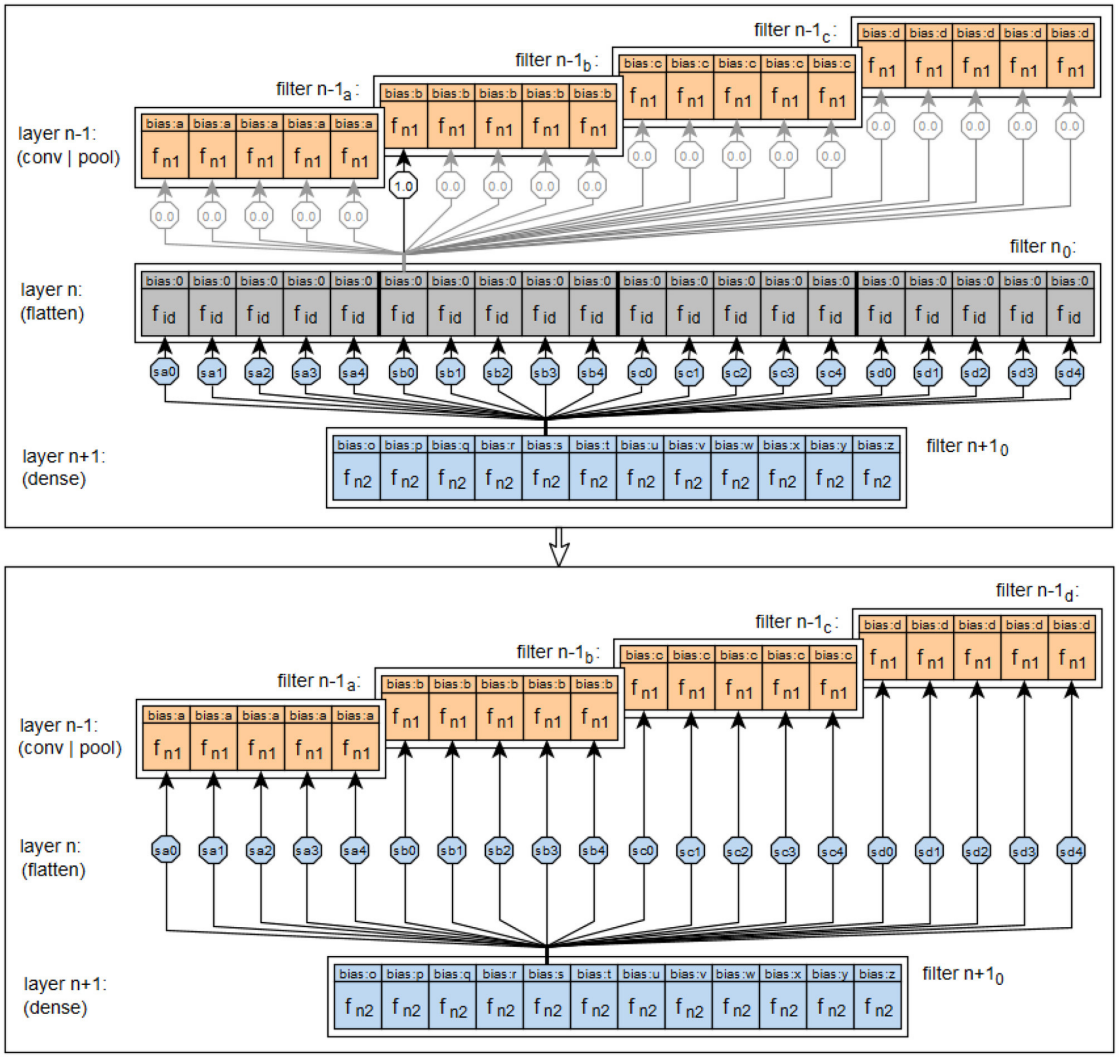
Visualization of the implementation of the principle of flattening layers in the prototype.

TensorFlow with Keras provides a number of layers that can be used to reshape the output of a previous layer to match a different, required input form of a subsequent layer. One such layer is the *flattening layer*. This layer converts its respective input data, usually a multidimensional tensor containing the output activations of the filters of a previous layer, into a one-dimensional output tensor, which speeds up the process later on. Figure 14 shows the implementation of the flattening layer functionality in the prototype. The upper part of the figure shows the connection structure that would be needed if flat layers were interpreted as feedforward layers with neurons. The lower part shows how the connection structure from the upper part can be summarized. As the figure shows, the prototype does not convert the flattening layers into a feedforward form, unlike the convolutional and pooling layers, although this would be possible. This is due to the function of the flattening layers, which only adjust the connections between their predecessor and successor layers. However, no new activation values are created or existing activation values are processed, so no new combinations are created by a flattening layer. Therefore the functionality of the flattening layers in the prototype is not realized by neurons, but in the methods of the classes NeuralNet and NeuralLayer, which are relevant for the connections between the layers.

### 4.3 The generation of hierarchical decision trees

The class TreeNode represents the necessary data structure for the realization of node elements in a hierarchical decision tree. When generating hierarchical decision paths, it was not yet necessary to explicitly create such structures, since in all decision levels an edge *e* is always followed by exactly one node *n* and each node *n* is followed by at most one edge *e*. So the vertices did not need to be considered explicitly. However, when combining multiple paths into a hierarchical decision tree, nodes are needed to carry information when creating the tree structure. Objects of type Merger each perform the procedure for combining their internal decision paths, recorded as TreePath, into a combined decision tree for an assigned TreeEdge. The result is the root node of the internal decision tree representing the assigned TreeEdge.

**Figure 15:**
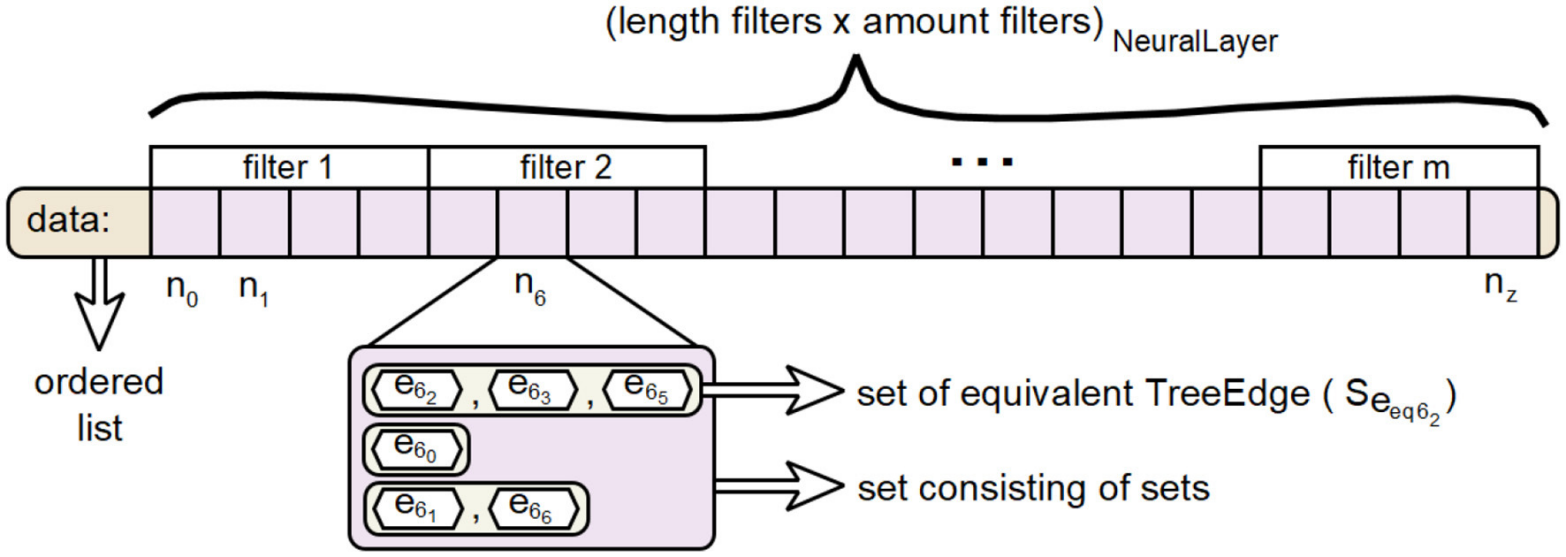
Visualization of the data structure behind the data attribute of the EquEdgesSet class.

A visualization of the EquEdgesSet class for the the data attribute is shown in Figure 15. This is an approach to perform the calculations for merging paths into a tree in the prototype without excessively inflating the heap.

## 5 Summary and conclusion

The following is a summary of the overall procedure to derive a hierarchical decision tree from a feedforward neural network and a set of input vectors given to the network. The structural information of the analyzed network is read in and, if it is not already in this form, converted into a complete feedforward form. This is necessary, for example, for convolutional networks, which correspond to feedforward networks with a special connection structure in the convolutional, pooling, and flattening layers. For the *n* layers of the network, starting with the first layer, the respective estimates for the upper bound of the maximum output activation and the lower bound of the minimum output activation are determined. Then, the potentially relevant weighted connections and thus the potentially relevant neurons of the examined network are determined from the output layer to the input layer in order to reduce the computational effort. For each of the input vectors to be examined, the following steps are performed:

1. the corresponding input vector is entered into the network and the resulting activations of all neurons for this input vector are calculated based on the minimized structure.
2. based on the calculated activations instead of the estimated boundaries, the relevant weighted connections and neurons of the minimized structure are calculated for the classification of the selected input vector. A *slider* for neural sensitivity is used to adjust the threshold θ for relevance regarding the weighted connections. Adjusting the threshold to a smaller value takes more of the weighted connections into account initially.
3. based on the connections and neurons relevant for the classification of the selected input vector, the hierarchical decision path for said classification can now be derived. The associated symbol equivalents for this decision path are also created. Again, the relevance threshold for this step can be adjusted in the prototype using the activation sensitivity slider. The higher the sensitivity value, the clearer the decision paths and the lower the accuracy.

The hierarchical decision paths that have been derived in the individual iterations are now combined to form a common hierarchical decision tree. The procedure described above has been implemented and tested in a prototype. This proved that the desired derivation of equivalent symbol-based decision models from feedforward neural networks can be efficiently implemented. In this way, the inner workings of feedforward neural networks can be shown, the black-box character can be broken down, and the opaque decisions can be traced using the resulting decision trees.

Currently, the methodology is tailored to feedforward and convolutional neural networks, which limits its applicability to architectures with static, layer-based structures. To extend the methodology to more complex models, such as recurrent neural networks (RNNs), one could unroll the temporal steps to approximate a feedforward form and incorporate memory states into symbolic representations. For transformer models, attention mechanisms could be translated into symbolic rules by tracing dominant attention paths. Extending to autoencoders or graph neural networks would require modeling latent or structural dependencies symbolically. While these extensions are conceptually feasible, they demand more sophisticated representations than decision trees, such as state machines or graph-based symbolic models. The effectiveness of the proposed methodology is also expected to depend on the specific architecture and training characteristics of the underlying neural network. We plan to investigate how the approach can be adapted systematically to different model types.

Furthermore, as the complexity and depth of a neural network increase, so does the computational effort required to derive an equivalent symbolic model. Deeper architectures involve more layers, neurons, and weighted connections, all of which must be analyzed for their relevance to each decision path. This results in an increase in potential symbolic combinations and activation traces. Additionally, recursively substituting subpaths in hierarchical decision trees adds overhead. Future implementations may require pruning strategies, parallel processing, or approximate methods to balance symbolic fidelity with practical efficiency and ensure scalability of large-scale models.

Finally, to improve the assessment of how well the derived decision trees replicate the original neural networks, future work should incorporate quantitative fidelity metrics. These metrics, such as prediction agreement and fidelity scores, would allow for a rigorous evaluation of symbolic accuracy and guide refinements to ensure faithful and interpretable approximations of complex models.

## Data Availability

Publicly available datasets were analyzed in this study. This data can be found here: https://github.com/SeSeidel/CalculatorDemonstrator/tree/main.

## Footnotes

^1^There are a few viable alternatives to replace the chosen equation. *Absolute thresholding* applies a fixed cutoff ε, offering simplicity but lacking adaptability. *Top-**K* filtering retains the most influential inputs, highlighting key contributors but potentially missing subtler effects (Montavon et al., 2018). *Percentage contribution* includes inputs exceeding a relative threshold θ, providing scale invariance at moderate computational cost. More advanced techniques include *Layer-wise Relevance Propagation* (LRP), which distributes output relevance scores across layers, and *SHAP/Integrated Gradients* (Lundberg and Lee, 2017; Sundararajan et al., 2017), which offer high-resolution attributions but with greater computational demands (Nyiri and Gibaru, 2025). Each method balances interpretability, precision, and performance differently.
